# 

**Published:** 1862-05

**Authors:** 


### CHICAGO MEDICAL JOURNAL.

**Terms, $2.00 per Annum.**

### CONTENTS OF THE MAY NUMBER.

ORIGINAL COMMUNICATIONS.

PAGE.

Nitric Acid in Whooping Cough. Read before the McLean County Medical Society, by S. W. Noble, M. D. .......... 253

Cases of Uræmia as Sequela of Scarlet Fever. By W. Brooks, M. D., of Chicago.......... 257

The Persulphate of Iron as an Agent for the Destruction of Polypus of the Ear. By E. L. Holmes, M. D., of Chicago.......... 267

Pittsburgh Landing.......... 271

A Case of Puerperal Convulsions in Charge of David Dodge, M. D., of Chicago.......... 281

SELECTED.

The Action of the Voluntary Muscles. By Louis Mackall, M. D.......... 285

Prosecution for Infanticide.......... 293

Influence of Climate on Phthisis.......... 306

Removing the Clitoris in Cases of Masturbation, accompanied with Threatening Insanity. By E. S. Cooper, A. M., M. D., Prof. of Anatomy and Surgery in the University of the Pacific.......... 307

EDITORIAL AND MISCELLANEOUS.......... 311

De Witt County Medical Society.......... 313

